# Platelet thromboxane inhibition by low‐dose aspirin in polycythemia vera: Ex vivo and in vivo measurements and in silico simulation

**DOI:** 10.1111/cts.13415

**Published:** 2022-10-05

**Authors:** Giovanna Petrucci, Alberto Giaretta, Paola Ranalli, Viviana Cavalca, Alfredo Dragani, Benedetta Porro, Duaa Hatem, Aida Habib, Elena Tremoli, Carlo Patrono, Bianca Rocca

**Affiliations:** ^1^ Department of Safety and Bioethics, Section of Pharmacology Catholic University School of Medicine Rome Italy; ^2^ Department of Pathology University of Cambridge Cambridge UK; ^3^ Department of Hematology S. Spirito Hospital Pescara Italy; ^4^ Centro Cardiologico Monzino, IRCCS Milan Italy; ^5^ Department of Basic Medical Sciences, College of Medicine, QU Health Qatar University Doha Qatar; ^6^ Maria Cecilia Hospital Cotignola Italy

## Abstract

Low‐dose aspirin is currently recommended for patients with polycythemia vera (PV), a myeloproliferative neoplasm with increased risk of arterial and venous thromboses. Based on aspirin pharmacodynamics in essential thrombocythemia, a twice‐daily regimen is recommended for patients with PV deemed at particularly high thrombotic risk. We investigated the effects of low‐dose aspirin on platelet cyclooxygenase activity and in vivo platelet activation in 49 patients with PV, as assessed by serum thromboxane (TX) B_2_ and urinary TXA_2_/TXB_2_ metabolite (TXM) measurements, respectively. A previously described pharmacokinetic‐pharmacodynamic in silico model was used to simulate the degree of platelet TXA_2_ inhibition by once‐daily (q.d.) and twice‐daily (b.i.d.) aspirin, and to predict the effect of missing an aspirin dose during q.d. and b.i.d. regimens. Serum TXB_2_ averaged 8.2 (1.6–54.7) ng/ml and significantly correlated with the platelet count (*γ* = 0.39) and urinary TXM (*γ* = 0.52) in multivariable analysis. One‐third of aspirin‐treated patients with PV displayed less‐than‐maximal platelet TXB_2_ inhibition, and were characterized by significantly higher platelet counts and platelet‐count corrected serum TXB_2_ than those with adequate inhibition. Eight patients with PV were sampled again after 12 ± 4 months, and had reproducible serum TXB_2_ and urinary TXM values. The in silico model predicted complete inhibition of platelet‐derived TXB_2_ by b.i.d. aspirin, a prediction verified in a patient with PV with the highest TXB_2_ value while on aspirin q.d. and treated short‐term with a b.i.d. regimen. In conclusion, one in three patients with PV on low‐dose aspirin display less‐than‐maximal inhibition of platelet TXA_2_ production. Serum TXB_2_ measurement can be a valuable option to guide precision dosing of antiplatelet therapy in patients with PV.

**Study Highlights**

WHAT IS THE CURRENT KNOWLEDGE ON THE TOPIC?

Polycythemia vera (PV) is a myeloproliferative neoplasm (MPN) characterized by clonal erythrocytosis and increased thrombotic complications. Once‐daily, low‐dose aspirin is currently recommended in patients with PV.

WHAT QUESTION DID THIS STUDY ADDRESS?

The aim of the present study was to characterize the antiplatelet pharmacodynamics (PDs) of low‐dose aspirin, its determinants, and the level of in vivo platelet activation in a contemporary PV cohort. We also used in silico modeling of aspirin pharmacokinetics/PDs to simulate the platelet response to a once‐daily regimen, and to predict the effects of a twice‐daily regimen on thromboxane (TX) production in PV.

WHAT DOES THIS STUDY ADD TO OUR KNOWLEDGE?

One in three patients with PV display inadequate inhibition of platelet TXA_2_ production and TXA_2_‐dependent platelet activation, despite standard aspirin therapy. In silico modeling may help design future studies in this setting.

HOW MIGHT THIS CHANGE CLINICAL PHARMACOLOGY OR TRANSLATIONAL SCIENCE?

Impaired aspirin PDs can be diagnosed with a serum TXB_2_ determination 24 h after dosing, and can be rescued by a b.i.d. dosing regimen. This relatively simple approach may be applied to other non‐MPN clinical settings.

## INTRODUCTION

Polycythemia vera (PV) is a Philadelphia‐negative, myeloproliferative neoplasm (MPN) characterized by clonal erythrocytosis and enhanced risk of arterial and venous thromboses affecting the quality and duration of life.^1^, ^2^, ^3^ The annual incidence of first major arterial thrombosis is as high as ~3% despite antiplatelet therapy.^1^, ^4^

The currently recommended antithrombotic strategies for PV include: (i) low‐dose aspirin (75–100 mg, once daily [q.d.]), and (ii) targeting an hematocrit value less than 45%, considered as the upper limit of the physiologic range,^5^ with phlebotomy and/or chemotherapy (mainly hydroxyurea [HU]).^5^ The European Collaboration on Low‐Dose Aspirin in Polycythemia Vera (ECLAP) trial tested the efficacy and safety of low‐dose aspirin versus placebo in 518 patients with PV.^6^ Aspirin reduced the risk of the composite end point of nonfatal myocardial infarction, stroke, pulmonary embolism, major venous thrombosis, or cardiovascular death (relative risk, 0.40; 95% confidence interval [CI], 0.18 to 0.91; *p* = 0.03), based on eight vascular events in the aspirin arm and 21 in the placebo arm.^6^ The CytoPV trial randomized 365 patients with PV to hematocrit values less than 45% or between 45% and 50%.^7^ The annual incidence of major thromboses or cardiovascular death was 1.1% and 4.4% in the low‐ and high‐hematocrit group, respectively, with ~85% of patients in both groups being on antiplatelet treatment, mostly low‐dose aspirin.^7^

Over the past 10 years, studies in a different MPN, essential thrombocythemia (ET), showed that a traditional regimen of low‐dose aspirin is unable to fully inhibit platelet cyclooxygenase (COX) activity in the majority of patients with ET, most likely because of the enhanced platelet renewal rate exceeding the capacity of a q.d. aspirin regimen to steadily inactivate platelet COX activity throughout the 24‐h dosing interval.^8^ Platelet inhibition in patients with ET progressively decreased between 12 and 24 h after aspirin dosing^1^, ^9^, ^10^ and was rescued by a twice‐daily (b.i.d.) regimen.^11^ Based on these pharmacodynamic (PD) data, a b.i.d. aspirin regimen was recently recommended for patients with PV deemed at “high” thrombotic risk based on Janus Kinase 2 (*JAK2*) *V617F* mutation allelic burden, leukocytosis, age greater than 60 years, and/or previous thrombosis.^5^, ^11^ However, at variance with ET, the pharmacokinetic (PK) and PDs of low‐dose aspirin in PV have not been adequately investigated.

The aim of the present study was to characterize the antiplatelet PD of low‐dose aspirin, its determinants, and the level of in vivo platelet activation in a contemporary PV cohort.^12^ We also used in silico modeling of aspirin PK/PD^9^ to simulate the platelet response to a q.d. regimen, and to predict the effects of a b.i.d. regimen on thromboxane (TX) production in PV. Furthermore, we tested this prediction in a phase I trial in a patient with PV with the highest TXB_2_ value while on aspirin q.d., who was treated short‐term with a b.i.d. regimen.

## MATERIALS AND METHODS

### Population under study

Forty‐nine consecutive patients with PV diagnosed according to the World Health Organization (WHO) 2016 criteria, on low‐dose aspirin from greater than or equal to 3 months according to current guidelines, were enrolled at the Santo Spirito Teaching Hospital in Pescara, between October 2017 and December 2019. Exclusion criteria were: smoking greater than five cigarettes/day; significant hepatic, renal, cardiac, or pulmonary insufficiency; cancer less than or equal to 5 years; poorly controlled hypertension or hypercholesterolemia; pregnancy; lactation; chronic use (>3 times/week) of nonsteroidal anti‐inflammatory drug (NSAID); treatment with anticoagulants or antiplatelet agents other than aspirin; a recent (<3 months) myocardial infarction, stroke or major bleeding event; congenital bleeding disorder; a platelet count less than 150 × 10^3^/μl, which is the lower limit of the normal range. Consenting patients underwent a run‐in phase of 7–9 days, during which aspirin intake (100 mg, enteric‐coated formulation, Cardioaspirin, Bayer, Italy) was synchronized at breakfast (7–9 a.m.) and NSAIDs were prohibited. Patients underwent a study visit 24 h after the last aspirin intake, and urine and blood samples were collected. Aspirin and NSAID intake were checked by telephone calls and written reminders during the run‐in, and through a patient interview at study visit. To assess the reproducibility of aspirin PDs, eight consenting patients with PV were studied with the same protocol between 8 and 16 months after the first visit. The patient with PV with the highest serum TXB_2_ value consented to being treated with aspirin 100 mg b.i.d. for 2 weeks, and a serum TXB_2_ measurement was repeated at the end of this experimental treatment. The study protocol and informed consent form were approved by the institutional ethics committee of the Santo Spirito Hospital of Pescara. All patients signed the informed consent. All procedures were conducted in accordance with the ethical standards of the 1975 Helsinki Declaration.

### Biomarker measurements

Routine hematochemistry was measured by standard procedures*. JAK2V617F* mutation was assessed on genomic DNA from peripheral blood mononuclear cells with a commercial kit (JAK2 MutaScreen RS kit; QIAGEN GmbH, Hilden, Germany).

Serum TXB_2_ was measured as an index of platelet COX activity,^13^ from venous blood samples collected without anticoagulant, clotted for 1 h at 37°C, and centrifuged at 1200 *g* for 10 min. Serum TXB_2_ was measured with a previously described enzyme immunoassay.^14^ Urinary 11‐dehydro‐TXB_2_ (TXM), an index of in vivo platelet activation,^15^ and 8‐iso‐prostaglandin (PG)F_2α_, an index of in vivo lipid peroxidation,^16^ were measured by previously described and validated immunoassays,^15^, ^16^ and expressed as pg per milligram of urinary creatinine, measured with a commercial kit (Creatinine Colorimetric Detection Kit; Enzo Life Sciences, Farmingdale, NY). Urinary 2,3‐dinor‐6‐keto‐PGF_1α_, a major enzymatic metabolite of PGI_2_ (PGIM), was measured by liquid chromatography–tandem mass spectrometry as previously described,^17^ using urine added with deuterated internal standard (d9‐PGI‐M), extracted with ethyl acetate, and analyzed by a triple quadrupole mass spectrometer (Quantum Access; Thermo Fisher Scientific, San Jose, CA). Plasma esterase activity and the degree of aspirin hydrolysis were measured in vitro by salicylic acid (SA) production over time, as previously described.^11^ The amount of hydrolyzed aspirin (equimolar to the amount of SA formed) was represented by the slope of the time curve, expressed as SA μmol/ml/min.

High‐sensitivity C‐reactive protein (hs‐CRP) and interleukin (IL)‐6 were measured with commercial kits (High‐sensitivity CRP ELISA Kit; Cyclex Co., Nagano, Japan; Interleukin‐6 ELISA Kit; Cayman Chemical Company, Ann Arbor, MI).

### In silico model of aspirin PKs/PD

In this section, we report the mathematical description of an in silico mathematical model of low‐dose aspirin previously detailed.^9^ A linear three‐compartment model with first‐order absorption, and 4‐h absorption lag time was utilized for the enteric‐coated aspirin PKs.^9^

The COX‐1 dynamics in a single megakaryocyte (MK) unit was modeled as follows. During the maturation phase, lasting *T*_*1*_, COX‐1 dynamics in a single MK unit of cells born at time *τ*_*i*_ is described as the balance between input (i.e., de novo synthesis) and output (i.e., degradation and acetylation) fluxes, while during the platelet generation an additional output flux describes COX‐1 transfer to platelets, lasting *T*_*2*_. Dynamic events, such as COX‐1 synthesis, degradation, and transfer to platelets, depend upon the lag between time *t* and time *τ*_*i*_, so that model equations are:

(1) $${\dot{x}}_{\mathrm{MK}}\left(t\right)=\left\{\begin{array}{ll} p\left(t-\tau_{i}\right)-k_{d,x}\left(t-\tau_{i}\right)\;x_{\mathrm{MK}}\left(t\right)-\varphi \left(\left[x_{\mathrm{MK}}\left(t\right)\right],\left[A_{T}\left(t\right)\right]\right) & \text{maturation}:t\in \left[\tau_{i},\tau_{i}+T_{1}\right]\\ p-k_{d,x}\;x_{\mathrm{MK}}\left(t\right)-k_{5}\left(t-\tau_{i}\right)\;x_{\mathrm{MK}}\left(t\right)-\varphi \left(\left[x_{\mathrm{MK}}\left(t\right)\right],\left[A_{T}\left(t\right)\right]\right) & \text{platelet generation}:t\in \left[\tau_{i}+T_{1},\tau_{i}+T_{1}+T_{2}\right] \end{array}\right.$$

where:

- *x*_*MK*_ *(t)* [ng] and [*x*_*MK*_ *(t)*] = *x*_*MK*_ *(t)*/ *v*_*MK*_ *(t)* [ng/ml] are the time course of COX‐1 amount and concentration, respectively, inside a generic MK unit originated at time *τ*_*i*_. The volume *v*_*MK*_ *(t)* was modeled using proper functions, to account for the exponential growth during maturation and the exponential decay during the platelet generation.^9^
- *p(t‐τ*_*i*_ *)* [ng/min] and *k*_*d,x*_ *(t‐τ*_*i*_ *)* [min^−1^] represent COX‐1 input function from expression/translation and degradation rate constant, respectively. They were phenomenologically modeled in order to reproduce a fast COX‐1 expression at the beginning of the megakaryocyte life, followed by a decline to a constant level in the remaining life of the megakaryocyte. In particular, *p(t‐τ*_*i*_ *)* is rescaled by a factor *f*_*1*_ that account for the pathological condition. Further details are presented in our original work.^9^
- $\varphi \left(\left[x_{\mathrm{MK}}\left(t\right)\right],\left[A_{T}\left(t\right)\right]\right)=\frac{\lambda {\left[\left[A_{T}\left(t\right)\right]\cdot \left[x_{\mathrm{MK}}\left(t\right)\right]\right]}^{n}}{{\left(k_{m}\right)}^{n}+{\left[\left[A_{T}\left(t\right)\right]\cdot \left[x_{\mathrm{MK}}\left(t\right)\right]\right]}^{n}}$ [ng/min] is COX‐1 acetylation by aspirin, taking place in the MK unit, modeled as a sigmoidal function of the product of COX‐1 and aspirin concentrations at time *t*, in keeping with a threshold‐saturation mechanism proposed in literature.
- $k_{5}\left(t-\tau_{i}\right)$ is the rate constant at which COX‐1 leaves the MK unit compartment and is transferred to platelets during the final maturation stage, expressed in terms of the reduction in MK unit volume *v*_*MK*_.

Thus, the COX‐1 dynamics in the platelets formed from a single MK unit result from the balance between the input flux from platelet generation, lasting *T*_*2*_ and present only in the early phase, and two output fluxes the former due to acetylation, lasting *T*_*3a*_, and the latter to peripheral platelet destruction lasting *T*_*3b*_, so that model equations are:

(2) $${\dot{x}}_{P}\left(t\right)=\left\{\begin{array}{ll} \;k_{5}\left(t-\tau_{i}\right)\;x_{\mathrm{Mk}}\left(t\right)-\varphi \left(\left[x_{p}\left(t\right)\right],0.75\left[A_{B}\left(t\right)\right]+0.25\left[A_{S}\left(t\right)\right]\right) & \text{platelet generation}:t\in \left[\tau_{i}+T_{1},\tau_{i}+T_{1}+T_{2}\right]\\ -\varphi \left(\left[x_{p}\left(t\right)\right],0.75\left[A_{B}\left(t\right)\right]+0.25\left[A_{S}\left(t\right)\right]\right) & \text{platelet activity}:t\in \left[\tau_{i}+T_{1}+T_{2},\tau_{i}+T_{1}+T_{2}+T_{3a}\right]\\ -\varphi \left(\left[x_{p}\left(t\right)\right],0.75\left[A_{B}\left(t\right)\right]+0.25\left[A_{S}\left(t\right)\right]\right)-k_{6}\left(t-\tau_{i}\right)x_{P}\left(t\right) & \text{platelet death}:t\in \left[\tau_{i}+T_{1}+T_{2}+T_{3a},\tau_{i}+T_{1}+T_{2}+T_{3a}+T_{3b}\right] \end{array}\right.$$

where:

- *x*_*p*_ *(t)* [ng] and [*x*_*p*_ *(t)*] = *x*_*p*_ *(t)/ v*_*p*_ *(t)*[ng/ml] are the time course of COX‐1 amount and concentration in platelets derived from a single MK unit. The volume *v*_*p*_ *(t)*, increases during MK proliferation, parallel to the decrease of MK volume, remains constant during platelet life, and then decreases to zero during their death phase.
- *φ*([*x*_*p*_ *(t)*]*,0.75*[*A*_*B*_ *(t)*] *+ 0.25*[*A*_*S*_ *(t)*]) [ng/min] is the sigmoidal COX‐1 acetylation function inside platelets modulated by a weighted sum of aspirin patterns in the systemic and portal blood, assuming a 75/25 ratio of systemic/portal blood volume.
- $k_{6}\left(t-\tau_{i}\right)$ [min^−1^] is the rate constant of COX‐1 disappearance, due to their peripheral destruction.

The time course of COX‐1 at whole‐body was obtained by summing up the contributions of all platelets in the systemic circulation, originating from MK units that were born at a time *τ*_*i*_ ranging from t‐T_1_‐T_3_ and t‐T_1_ where T_1_ and T_3_ are the MK and platelet lifetime, respectively. This variable, denoted by X(t), can be expressed as:

(3) $$X\left(t\right)=\sum_{\tau_{i}\in \left[t-T_{1}-T_{3},\;t-T_{1}\right]}x_{p}\left(t-\tau_{i}\right)$$

where x_p_ represents the amount of COX‐1 inside the platelets derived from the MKs born at a time *τ*_*i*_.

The whole‐body COX‐1, X(t), is then converted into a variable T(t) that accounts for the whole‐body serum TXB_2_ and can be expressed as:

(4) $$T\left(t\right)=g\left(X\left(t\right)\right)$$

Where *g*() is an experimentally derived nonlinear function previously described.^9^

### Evaluation of the original model of low‐dose aspirin in healthy subjects and patients with ET

The calibration and evaluation of the above model has been previously detailed.^9^ Briefly, few key parameters were tuned to fit the experimental data (in particular that related to COX‐1 acetylation, COX‐1 biosynthesis, and the lifespan of MKs and platelets), whereas most parameters were inferred from literature (as it is deeply detailed in our original work),^9^ for example, PK parameters (derived by considering nominal values of aspirin clearance, blood flows and volumes), the physiological range of MK and platelet lifespan, the range of platelet count, and the COX‐1 degradation. In particular, model parameters were inferred from the literature and calibrated using measurements of serum TXB_2_, as a proxy of COX‐1 activity in peripheral platelets, in three different datasets. In particular, the model was calibrated to predict the average of subjects among the clinical scenarios of interest. The model was first evaluated on a dataset of time series of serum TXB_2_ from 21 healthy subjects treated with 100 mg aspirin.^9^, ^18^ Then, starting from a healthy condition, the model was further calibrated on serum TXB_2_ time series from patients with ET studied with increasing aspirin doses (i.e., 50, 100, and 150 mg) during and following aspirin treatment.^8^, ^9^, ^19^ Finally, the model under ET condition was refined and evaluated with a third dataset of 22 patients with ET under different aspirin regimens (i.e., 100 and 200 mg).^8^, ^9^ The parameters re‐calibrated to adapt the model to the ET conditions were those related to aspirin PDs (i.e., the parameter *λ* related to the COX‐1 acetylation, and the parameters related to the thrombopoiesis mechanism, i.e., the parameters related to the lifespan of MKs and platelets, the COX‐1 biosynthesis rate and the platelet count).^9^

### Calibration of the model for the patients with PV

In this work, we consider a model calibrated in healthy and ET subject conditions^9^ to start the model's re‐calibration for the current PV datasets. Given that the experimental results (described in the Results, Supplementary Information, and Figures S1‐S3) of the subset of patients with PV with serum TXB_2_less than or equal to 10 ng/ml were comparable based on confidence interval defined by mean ± SD or 25th‐75th interquartile, to the healthy subject's PD in the original model,^9^ we made the assumption to start with a model condition calibrated on the healthy subjects,^9^ as reported in the previous paragraph. Given the experimental results and the a priori knowledge of PV disease, it was reasonable to assume that the COX‐1 acetylation parameters, as well as the parameters related to thrombopoiesis (e.g., lifespan of MKs and platelets) remained unaltered compared to the healthy subject calibration. Platelet count was set to 1.4‐fold increase (compared to the healthy condition averaging ~250 × 10^3^/μl) consistent with the PV experimental data. Therefore, the model was re‐calibrated on the PV dataset, in order to predict the average of PV subjects, in terms of the best‐fit adjustment of the only COX‐1 biosynthesis parameter that was found to be 1.8‐fold increase compared to the COX‐1 biosynthesis value, calibrated on the healthy subject's dataset in the original model version.^9^ An increased value of COX‐1 biosynthesis was expected, as in ET condition in the original model,^9^ being both PV and ET MPN.

Given that data from the subset of patients with PV with serum TXB_2_greater than 10 ng/ml were associated with a higher platelet count and being both PV and ET MPN, it was reasonable to assume the original model version tuned on the ET datasets^8^, ^9^, ^19^ as a starting point. MK lifespan was set to 8 days in line with recently published studies in MPN^20^, ^21^ and its predictive consistency with the ET datasets reported in the previous paragraph was verified. As similarly done in the previous subpopulation of patients with PV, we made the reasonable assumption that the acetylation parameters, as well as the parameters related to thrombopoiesis (e.g., lifespan of MKs and platelets) remained unchanged as compared to the ET setup.^9^ In such a condition, we made a further assumption that COX‐1 biosynthesis could be altered compared to the healthy condition, in a comparable way to ET, being both MPN.^5^, ^20^

Platelet count was set to 1.7‐fold increase consistently with the PV laboratory data. Subsequently, the model was calibrated, to predict the average of PV subjects, to the new PV datasets in terms of the best‐fit adjustment of the COX‐1 biosynthesis parameter that was found to be 3.2‐fold increase compared to the COX‐1 biosynthesis value calibrated on the healthy subjects in the original model version.^9^ An increased value of COX‐1 biosynthesis was expected, as found in ET condition being both PV and ET MPNs. Moreover, it was expected to obtain a COX‐1 biosynthesis value for patients with PV with the serum TXB_2_ greater than 10 ng/ml higher than the value found for the serum TXB_2_ less than or equal to 10 ng/ml condition.

As for previous studies,^9^, ^22^ model predictions were normalized to a mean pre‐aspirin serum TXB_2_ of 575 ng/ml, based on previous studies from our group in two different cohorts of aspirin‐naïve patients with PV.^23^

### Evaluation of the model adapted for patients with PV

To evaluate our re‐calibrated model before running it to the cohort of the present study, we considered other two datasets. First, the original model was re‐calibrated on a dataset of non‐PV patients at high cardiovascular risk,^23^ which can be considered as patients having a normal bone marrow function and hence a physiological thrombopoiesis.^9^ Because these patients are similar to the healthy subjects in terms of thrombopoiesis, as indicated by their peripheral platelet count within the normal range, we used the original model^9^ calibrated on the healthy subjects as a starting point. It was reasonable to assume that the COX‐1 acetylation parameters, as well as the parameters related to thrombopoiesis (e.g., lifespan of MKs and platelets) remained unchanged, in fact, the platelet count was within the normal range averaging ~250 × 10^3^/μl. Therefore, the model was re‐calibrated on the non‐PV high cardiovascular risk dataset, in order to predict the average of subjects with PV, by re‐calibrating the only COX‐1 biosynthesis parameter that was found to be 1.5‐fold increase as compared to the COX‐1 biosynthesis value, calibrated on the healthy subject's dataset in the original model.^9^ Such value is similar (as confidence interval defined by mean ± SD or 25th‐75th interquartiles) to the value found for patients with PV with serum TXB_2_less than or equal to 10 ng/ml, thus reinforcing and evaluating the hypothesis that such responsive patients with PV are indeed close to the physiological and expected aspirin response.

Second, in order to effectively perform a first preliminary model evaluation on the patients with PV with serum TXB_2_greater than 10 ng/ml, we tested our re‐calibrated model (without altering any parameters) on a different, previously published dataset^23^ of serum TXB_2_ evaluated at 24 h after aspirin in patients with PV on aspirin 40 mg q.d. Table S1 shows the most relevant model parameters. Parameters with a clinical/biological role used for the current work are reported in Table S1.

### Calibrated model for patients with PV as in silico prediction tool

The model was used to simulate TXA_2_ inhibition in platelets^24^ from patients with PV on a b.i.d. aspirin regimen expressed as percentage of the level off aspirin (defined as “limit” value) and it was also used to predict the effect of missing an aspirin dose during q.d. and b.i.d. regimens.

### Statistical analyses

A serum TXB_2_ level less than or equal to 10 ng/ml was considered as the upper limit of optimal platelet inhibition in non‐MPN subjects based on the following: (i) this concentration represents greater than 97% reduction of pre‐aspirin serum TXB_2_ in aspirin‐treated healthy subjects^18^; and (ii) it corresponds to the upper limit of serum TXB_2_ measured 24 h after the last aspirin intake in non‐diabetic patients on low‐dose aspirin for cardiovascular prophylaxis.^25^ Based on previous data, we estimated that a sample size of 40 patients with PV would be required to detect a mean difference in serum TXB_2_ of at least 7 ng/ml versus aspirin‐treated non‐diabetic patients,^25^ 24 h after aspirin dosing, with a two‐tailed alpha of 0.05 and 99% power. Considering a potential 20% dropout, at least 48 patients had to be recruited.

All variables were tested for normal distribution, and non‐normally distributed variables were log‐transformed. Statistically significant values (*p* < 0.05) were used as independent variables, and analyses were performed by SigmaPlot software (version 13.0). Data are expressed as median and interquartile range, or mean and SD, as indicated.

## RESULTS

### Aspirin pharmacodynamics

Forty‐nine consecutive patients with PV (age 66 ± 11 years, 16 women) on treatment with low‐dose aspirin (100 mg, q.d.) for greater than or equal to 3 months participated in this study. The hematological and clinical characteristics as well as their correlations are detailed in Table 1, Supplemental Results, and Table S2, respectively.

**TABLE 1:** Main clinical and hematological characteristics of 49 patients with PV

Characteristic	Value
Age, years	67 [49–83]
Females, *n* (%)	16 (33)
Years since diagnosis	4 [0.25–17]
Years on aspirin	6 [0.4–18]
Hemoglobin, g/dl	13.8 [12–15.65]
Hematocrit, %	44 [37–49]
Platelets, ×10^3^/μl	344 [177–770]
White blood cells, ×10^3^/μl	8.14 [3.98–2.1]
Neutrophils, %	68 [55–85]
*JAK2V617F* allele burden, %	58 [13–95]
Hydroxyurea, *n* (%)	38 (77)
Phlebotomies, *n* (%)	36 (73)
Phlebotomy and hydroxyurea, *n* (%)	24 (49)
Type of thrombosis (number of episodes)	Deep vein thrombosis (4) Portal vein thrombosis (1) Acute coronary syndromes (7) TIA (2) Peripheral arterial occlusion (2)
Other comorbidities (*n*)	Diabetes under treatment (3) Medically controlled hypertension (18) Medically controlled hypercholesterolemia (5) Hypothyroidism under treatment (1)
Other comedications (*n*)	Proton pump inhibitors (9) Allopurinol (20)

*Note*: Data are expressed as median and [interquartile range].

Abbreviations: PV, polycythemia vera; TIA, transient ischemic attack.

In order to evaluate the adequacy of platelet COX‐1 inactivation by the standard regimen of low‐dose aspirin therapy in patients with PV, we measured platelet TXA_2_ production in response to endogenously formed thrombin during whole blood clotting ex vivo.^13^ The median serum TXB_2_ concentration was 8.2 (95% CI, 1.6–54.7) ng/ml, a value approximately three‐fold higher than that measured in low‐dose aspirin‐treated healthy subjects^23^ and non‐diabetic patients at high cardiovascular risk.^24^

**FIGURE 1:**
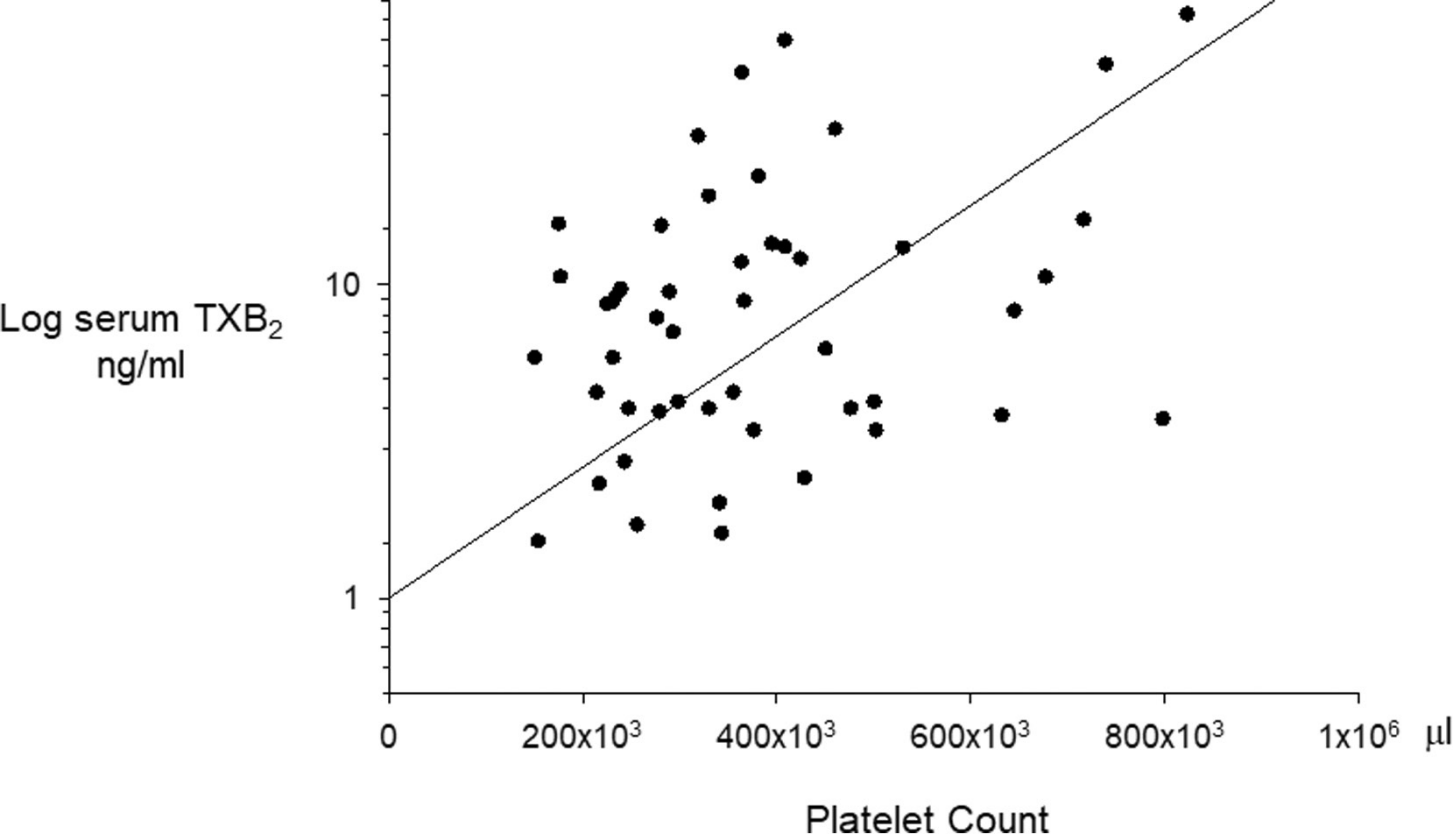
Correlation between platelet count and serum TXB_2_ in aspirin‐treated patients with polycythemia vera (PV). The figure shows the correlation between individual platelet counts and log serum TXB_2_ (ng/ml) in 49 patients with PV. TX, thromboxane

By univariate analysis, serum TXB_2_ significantly correlated with the platelet count (*γ* = 0.39, *p* = 0.005; Figure 1), whereas there was a weaker correlation with leukocyte (*γ* = 0.33, *p* = 0.02), and erythrocyte (*γ* = 0.34, *p* = 0.01) counts. By multivariable analysis, serum TXB_2_ was independently associated with the platelet count (adjusted *r* ^2^ = 0.19, *p* = 0.02). Given the wide range of platelet counts (151–852 × 10^3^/μl), we corrected serum TXB_2_ concentrations by the platelet count and expressed serum TXB_2_ as pg/platelet. Platelet count‐corrected serum TXB_2_ was approximately two‐fold higher in PV as compared to non‐PV patients (24 [95% CI, 10–39] vs. 13 [95% CI, 7–19] pg/platelet, respectively, *p* < 0.001).

**FIGURE 2:**
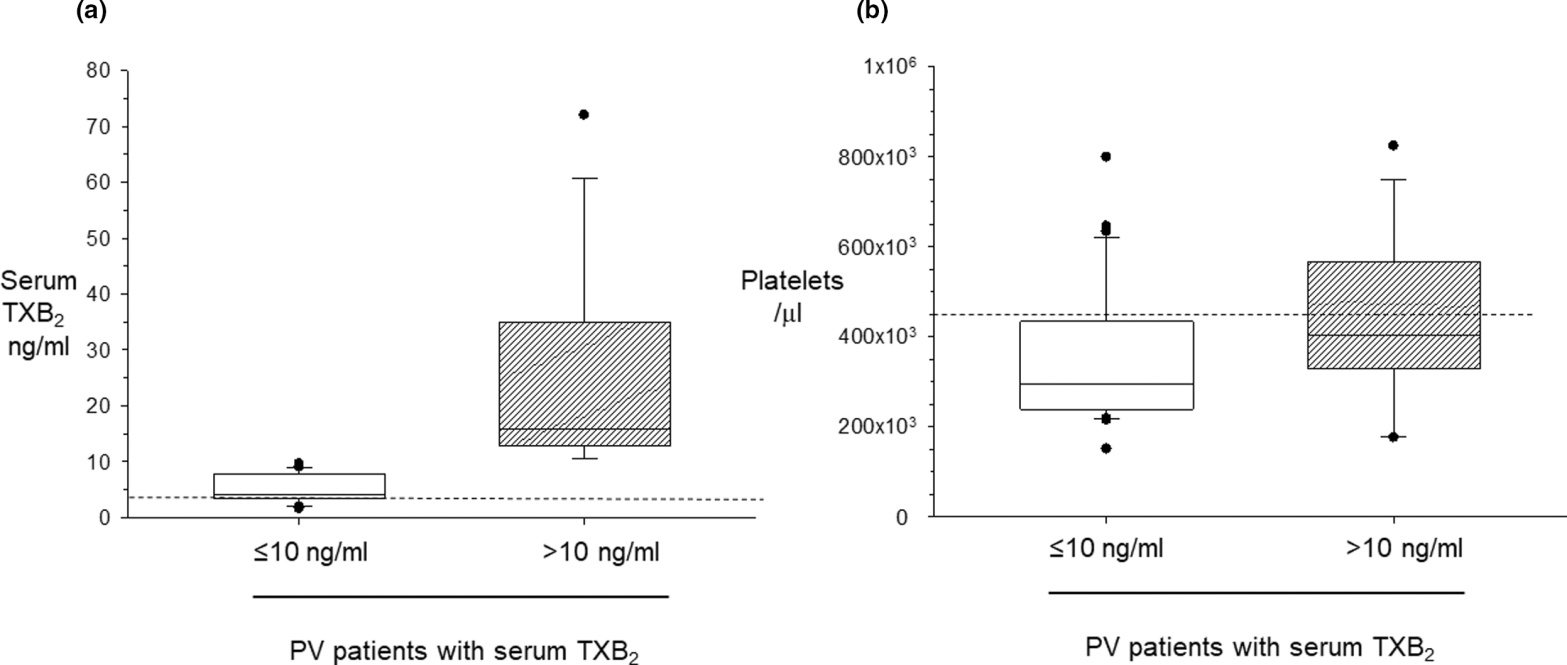
Median serum TXB_2_ and platelet counts in patients with polycythemia vera (PV) according to the (in)adequacy of platelet TXA_2_ suppression. Patients were grouped according to a threshold of serum TXB_2_ less than or equal to 10 ng/ml, corresponding to greater than 97% suppression of platelet cyclooxygenase‐1 activity. The box plots show the median values of serum TXB_2_ (a) and platelet counts (b) in each group. The dotted line in panel a indicates the median value in healthy subjects,^18^ and in panel b represents the upper limit of the laboratory for the normal population. TX, thromboxane

**FIGURE 3:**
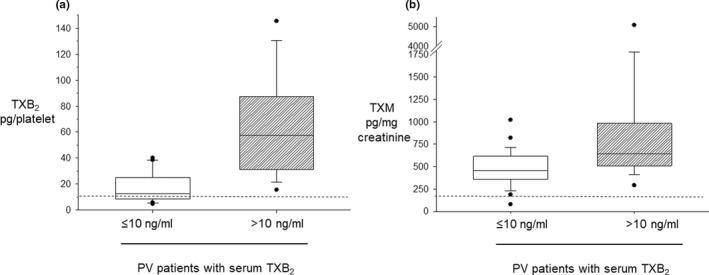
Median serum TXB_2_ normalized per platelet count and urinary TXM excretion in patients with polycythemia vera (PV) according to the 10 ng/mL threshold. Patients were grouped according to a threshold of serum TXB_2_ less than or equal to 10 ng/ml, corresponding to greater than 97% suppression of platelet cyclooxygenase‐1 activity. The box plots show the median values of serum TXB_2_ corrected per platelet count (panel a) and of urinary TXM (panel b) in each group. The dotted line in each panel indicates the median values in healthy subjects. TX, thromboxane; TXM, urinary TXA_2_/TXB_2_ metabolite

By using a 10 ng/ml upper limit of serum TXB_2_ concentrations to assess the adequacy of platelet TXA_2_ suppression by aspirin,^23^, ^24^ about two‐thirds of patients with PV had an appropriate level of platelet COX‐1 inhibition. As compared with patients with serum TXB_2_less than or equal to 10 ng/ml, patients with serum TXB_2_greater than 10 ng/ml (Figure 2a) had significantly higher platelet counts (Figure 2b). When analyzed as platelet count‐corrected serum TXB_2_, an approximately four‐fold difference was observed between the two groups (Figure 3a). In addition, patients with serum TXB_2_less than or equal to 10 ng/ml were more frequently on HU (81% vs. 66%, respectively), and had a lower incidence of previous thrombosis (7 out of 31 vs. 6 out of 18, respectively, *p* < 0.05) as compared with patients with serum TXB_2_greater than 10 ng/ml. Additional hematological and biomarker values in the two populations are detailed in Table S3.

Urinary TXM excretion, a noninvasive index of in vivo platelet activation,^26^ averaged 509 (95% CI, 205–1312) pg/mg creatinine, an excretion rate approximately four‐fold and two‐fold higher than previously reported in low‐dose aspirin‐treated healthy subjects,^18^ and patients with type‐2 diabetes mellitus,^25^ respectively, most likely reflecting a higher rate of platelet TXA_2_ biosynthesis in aspirin‐naive patients with PV than in the latter conditions.^26^ Urinary TXM was significantly correlated with serum TXB_2_, expressed both as ng/ml (*γ* = 0.52, *p* < 0.001) and as pg/platelet (*γ* = 0.58, *p* < 0.002), and with urinary 8‐iso‐PGF_2α_ (*γ* = 0.44, *p* = 0.002), a biomarker of in vivo lipid peroxidation.^16^ By multivariable analysis, serum TXB_2_/platelet (*p* < 0.001) and urinary 8‐iso‐PGF_2α_ (*p* = 0.009) were independently associated with urinary TXM excretion (adjusted *r* ^2^ = 0.41 for the entire model, each *p* < 0.01). Moreover, urinary TXM excretion was significantly higher in patients with serum TXB_2_greater than 10 ng/ml than in patients with values less than or equal to 10 ng/ml, suggesting that inadequate platelet COX‐1 inhibition was associated with higher platelet activation in vivo (Figure 3b).

Urinary PGIM excretion, an index of vascular PGI_2_ biosynthesis in vivo,^27^ averaged 219 ± 22 pg/mg creatinine, and was comparable to the levels previously reported in low‐dose aspirin‐treated healthy subjects.^28^ Urinary PGIM values were significantly correlated with urinary 8‐iso‐PGF_2α_ excretion, by both univariate and multivariable (*p* = 0.014) analyses.

Plasma esterase activity averaged 62 ± 10 μmol SA/L/min and were not different from levels measured in plasma of aspirin‐treated healthy subjects of comparable age (59 ± 30, *p* = 0.35 vs. patients with PV, Cavalca V., unpublished data), and was inversely correlated with age, both on univariate (*γ* = 0.42, *p* = 0.01) and multivariable analysis (*p* = 0.01).

Hs‐CRP values averaged 1.1 (95% CI, 0.26–16.23) μg/ml, not dissimilar from 50 age‐ and sex‐matched non‐PV patients on aspirin prophylaxis^25^ (2.3 [95% CI, 0.6–4.8] μg/ml, *p* = 0.10), and did not correlate with any of the variables under study (Table S2). IL‐6 averaged 3.9 (95% CI, 0.001–64.3) pg/ml (Table S2).

Eight patients with PV with stable disease and no complications were re‐studied after 12 ± 4 months. Their hematological parameters were comparable among the two study visits (Table S4, all *p* = ns). Serum TXB_2_ averaged 7.4 (95% CI, 4.2–10.2) and 8.8 (95% CI, 2.1–21.5) ng/mL on the first and second visits, respectively (*p* = 0.46 for paired comparison). Urinary TXM excretion rates averaged 505 (95% CI, 349–700) and 536 (95% CI, 394–750) pg/mg creatinine on the first and second visits, respectively, (*p* = 0.64 for paired comparison). Urinary 8‐iso‐PGF_2α_ was also stable over time (960 [95% CI, 714–1497] vs. 1017 [95% CI, 685–2115] pg/mg creatinine on the first and second visits, respectively; *p* = 1.0 for paired samples).

### In silico modeling, simulations, and predictions

**FIGURE 4:**
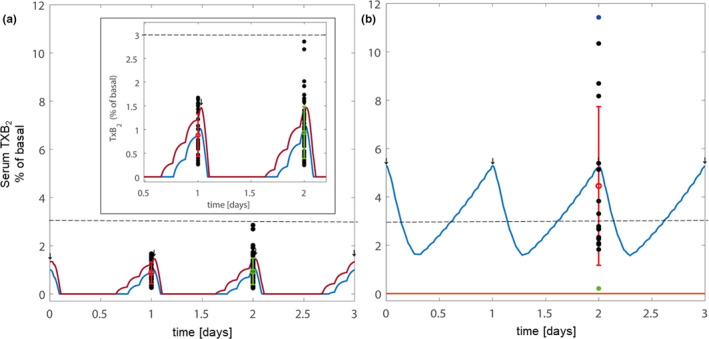
PK/PD in silico model predictions of serum TXB_2_ during chronic low‐dose aspirin treatment in patients with or without polycythemia vera (PV). (a) The in silico predictions were performed for 3 representative days under long‐term 100 mg once daily aspirin regimen for non‐PV patients (purple line) and patients with PV with adequate (i.e., ≤10 ng/ml, serum TXB_2_ suppression [blue line]). The experimental PV data, expressed as percentage of the mean value of PV patients off aspirin (“basal”),^24^ are also shown: the red dot and error bar represent the mean serum TXB_2_ (±SD) of 31 patients with PV with serum TXB_2_ less than or equal to 10 ng/ml. The green dot and error bar represent the mean serum TXB_2_ (±SD) expressed as percentage of off aspirin (“basal”) values, of 57 matched non‐PV patients.^25^ Black dots represent the overlaid TXB_2_ measurements related to each patient. (b) The in silico predictions of aspirin PK/PD were performed for 3 representative days under long‐term 100 mg once‐daily (blue line) and twice‐daily (red line) aspirin regimens in patients with serum TXB_2_ greater than 10 ng/ml. The experimental PV data of serum TXB_2_, expressed as percentage of the mean value of patients with PV off aspirin (basal),^24^ are also shown: the red dot and error bar represents the mean serum TXB_2_ (±SD) of 18 patients with PV with inadequate inhibition. The blue and green dots represent the serum TXB_2_ values of one compliant patient with PV with the highest serum TXB_2_ during treatment with aspirin 100 mg once daily and after 2 weeks of aspirin 100 mg twice‐daily, respectively. Black dots represent the overlaid TXB_2_ measurements related to each patient. All serum TXB_2_ values in the model are calculated and represented as percentage of the mean value of patients with PV off aspirin (“basal”).^24^ The dotted lines represent the threshold of 3% of basal serum TXB_2_ levels, corresponding to the steady, maximal platelet inhibition achieved in healthy subjects.^18^ PD, pharmacodynamics; PK, pharmacokinetics; TX, thromboxane

The in silico model was initially calibrated with the serum TXB_2_ and platelet datasets of patients with PV with serum TXB_2_less than or equal to 10 ng/ml, as well as with a dataset of non‐PV patients at high cardiovascular risk (Figure 4a), as detailed in the Methods section. Under both conditions, the experimental data and in silico simulations were remarkably consistent, suggesting that patients with PV with serum TXB_2_less than or equal to 10 ng/ml respond to aspirin treatment similarly to non‐PV patients with normal bone marrow function. A quantitative comparison between data and model predictions is reported in Table S5.

**FIGURE 5:**
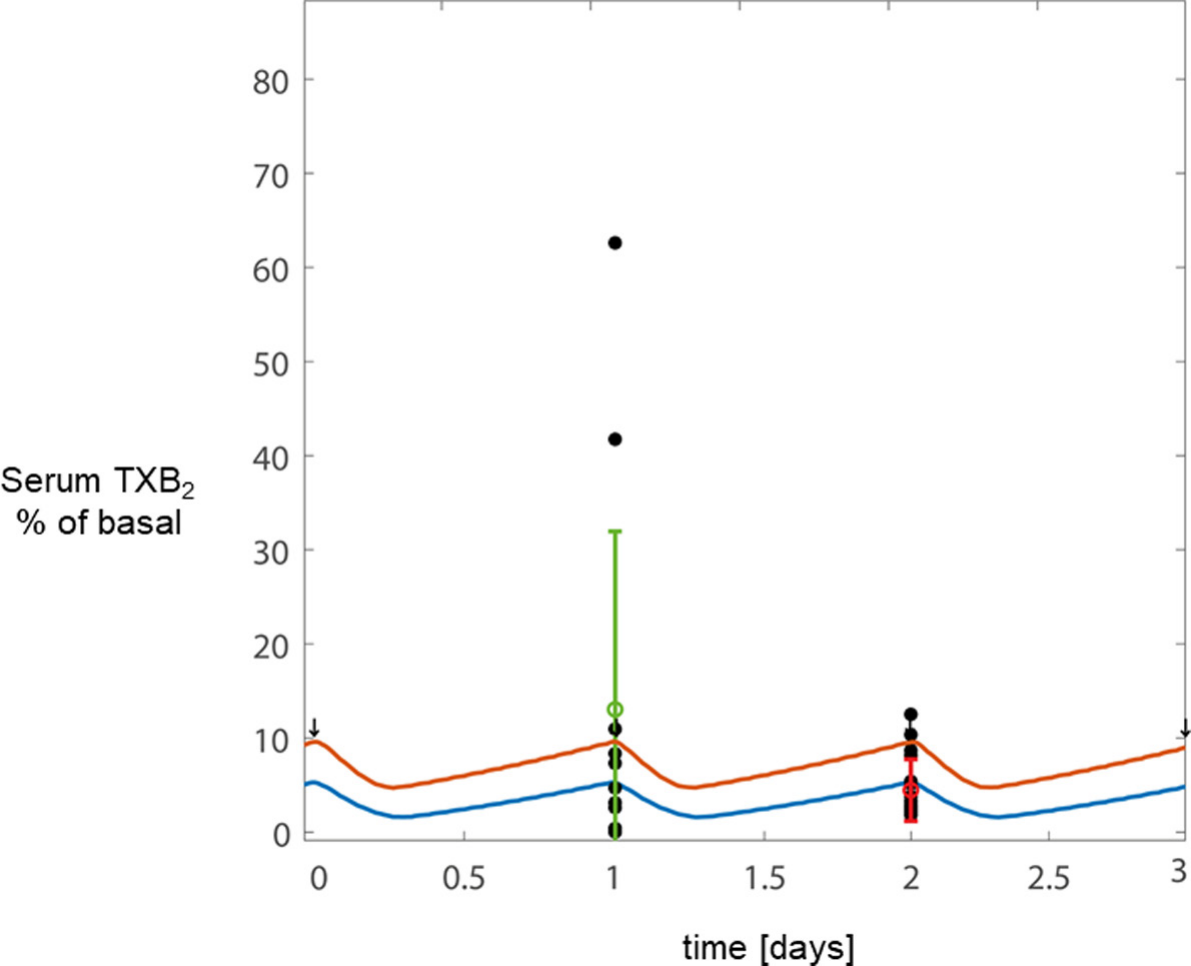
PK/PD in silico model predictions of serum TXB_2_ during chronic aspirin treatment in patients with polycythemia vera (PV) with serum TXB_2_ greater than 10 ng/ml on 100 and 40 mg q.d. The in silico predictions of aspirin PK/PD were performed for 3 representative days under long‐term 100 mg (blue line) and 40 mg (red line) q.d. aspirin regimens. The experimental PV data of serum TXB_2_, expressed as percentage of the mean value of patients with PV are also shown: the red dot and error bar represents the mean serum TXB_2_ (±SD) of 18 patients with PV with inadequate inhibition under 100 mg q.d., and the green dot represents serum TXB_2_ values of 10 patients with PV with inadequate inhibition under 40 mg q.d. Black dots represent the overlaid TXB_2_ measurements related to each patient. All serum TXB_2_ values in the model are calculated and represented as percentage of the mean value of patients with PV off aspirin.^23^ PD, pharmacodynamics; PK, pharmacokinetics; TX, thromboxane

To model and simulate serum TXB_2_ data of patients with PV with inadequate platelet TXA_2_ inhibition, we used the in silico model of aspirin PK/PD with parameters set to simulate the ET condition, an MPN characterized by high platelet counts and inadequate platelet TXA_2_ inhibition, thus mimicking patients with PV with serum TXB_2_greater than 10 ng/ml.^9^ Moreover, some crucial parameters specifically related to the PV condition were adjusted to accurately predict the actual serum TXB_2_ data of patients with PV (Figure 4b). In particular, the COX‐1 biosynthetic rate of the model and simulation was re‐calibrated to an ~3.2‐fold increase as compared to the value previously used for modeling data from healthy subjects.^9^ A preliminary model evaluation was performed by testing our re‐calibrated model on a previously published PV dataset,^23^ of 20 patients treated with aspirin 40 mg q.d., as a test set. Remarkably, the re‐calibrated model was capable to accurately predict the response of patients with PV in this previous PV dataset (Figure 5), hence reinforcing the robustness behind the PV‐tuned model. Further details about the adjustment of the model parameters are reported in the Methods section. Then, the model generated for patients with PV with inadequate serum TXB_2_ inhibition was used to predict the effect of a bid regimen as compared to the standard q.d. aspirin regimen (Figure 4b). The model predicted that the b.i.d. regimen would cause a complete and steady inhibition of serum TXB_2_ throughout the dosing interval (Figure 4b).

Based on the in silico prediction, the patient with PV with the highest serum TXB_2_ value while on aspirin 100 mg q.d., was treated with aspirin 100 mg bid for 2 weeks, and the patient's serum TXB_2_ was reduced from 50 to 1.3 ng/ml (i.e., to a level comparable with aspirin‐treated healthy subjects).^18^ These experimental data were qualitatively consistent with the in silico prediction (Figure 4 and Table S5).

**FIGURE 6:**
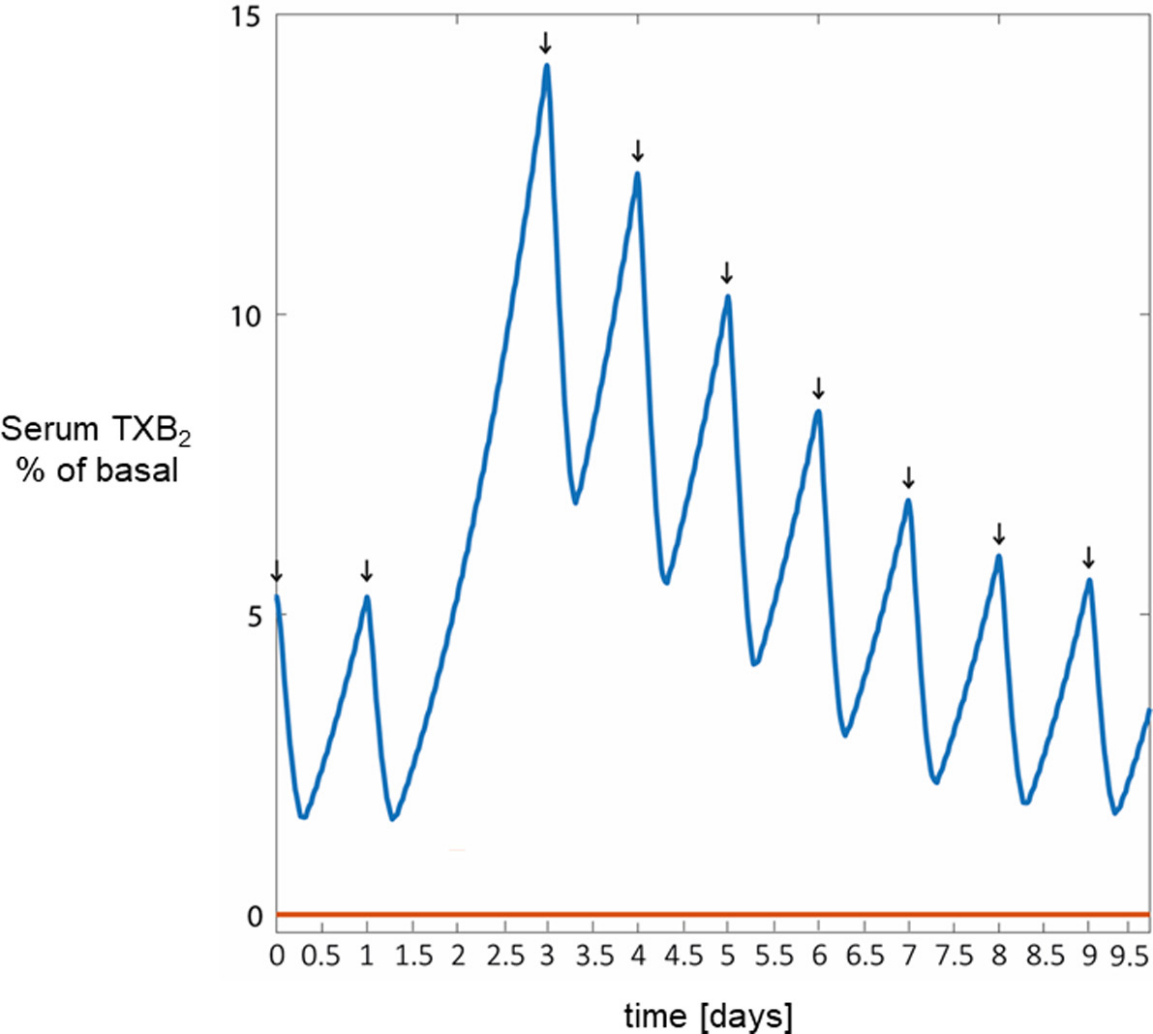
PK/PD in silico model predictions of serum TXB_2_ before and following a missed aspirin dose. The figure shows PK/PD in silico model predictions of serum TXB_2_ during chronic aspirin treatment in patients with PV poorly responsive to aspirin under a missing dose scenario. The in silico prediction of serum TXB_2_ considers the case of a patient with serum TXB_2_ greater than 10 ng/ml missing one 100‐mg aspirin dose at a representative day (day 2) under a once‐ (upper blue line) and twice‐ (lower red line) daily aspirin regimens. All serum TXB_2_ values in the model are calculated and represented in the plots as percentage of the mean value of patients with PV off aspirin, defined as “basal.”^24^ The arrows represent the time of intake of aspirin tablets. PD, pharmacodynamics; PK, pharmacokinetics; PV, polycythemia vera; TX, thromboxane

Finally, we used the in silico model to simulate and predict the kinetics of serum TXB_2_ recovery after missing one aspirin dose in patients with PV with inadequate TXA_2_ suppression, while being on the q.d. versus the b.i.d. regimen. Missing one aspirin dose while being on the q.d. regimen caused a rapid (24 h) and large (~3‐fold) increase in serum TXB_2_, which took 5 days to return to the level before the missed dose. On the contrary, no major fluctuations in serum TXB_2_ were predicted by the model in the case of missing one aspirin dose while being on the b.i.d. regimen (Figure 6).

## DISCUSSION

The present study thoroughly investigated platelet COX‐1 inhibition by a standard q.d. regimen of low‐dose aspirin, its stability over time, and its functional read‐out, as reflected by in vivo platelet activation, in a contemporary PV cohort, by means of a validated biomarker of aspirin PDs (i.e., TXB_2_ production during whole blood clotting).^12^

Previous studies used various platelet function (aggregometry‐based) assays, with arbitrary thresholds of aspirin “responsiveness” or “resistance” and included patients with different MPNs, with largely inconsistent results.^29^, ^30^, ^31^, ^32^, ^33^ At variance with aggregometry‐based assays, serum TXB_2_ specifically reflects the maximal biosynthetic capacity of platelet COX‐1, and this analytical approach has been instrumental in characterizing the human pharmacology of platelet COX‐1 inhibition in health and disease.^34^ In fact, important features of aspirin PD were revealed by serum TXB_2_ measurements: (i) the cumulative nature of platelet COX‐1 inactivation by repeated daily low doses; (ii) the saturability of this effect with single doses as low as 100 mg and repeated daily doses as low as 30 mg; and (iii) the COX‐isozyme selectivity of this inhibitory effect.^35^ A previous study assessed serum TXB_2_ in 19 patients with PV treated with aspirin 40 mg q.d., and reported a mean value of 25 ± 18 ng/ml, suggesting largely inadequate platelet COX‐1 inactivation by such an aspirin regimen.^23^

At variance with previous studies in ET, a different MPN in which ≈80% of patients had inadequate platelet inhibition while on a standard q.d. aspirin regimen,^1^, ^11^ only approximately one‐third of patients with PV displayed a similar phenotype, as defined by serum TXB_2_ values greater than 10 ng/ml. These patients with PV were characterized by a significantly higher platelet count, a trend toward higher red blood cell (RBC) and white blood cell (WBC) counts, and higher TXA_2_ biosynthetic rate per platelet, thus suggesting that the reduced extent of platelet inhibition by aspirin does not simply reflect a quantitative increase in the platelet mass, but also qualitative changes in the recovery of the platelet biosynthetic capacity during the 24‐h dosing interval. Moreover, the higher platelet TXA_2_ production in this subgroup was significantly associated with the rate of in vivo platelet activation, as reflected by urinary TXM excretion (Figure 3b).^26^ This association has been recently reported in aspirin‐treated patients with ET,^12^ suggesting that a reduced antiplatelet effect of aspirin may be responsible for in vivo platelet activation and plausibly translate into increased thrombotic risk.^1^ High urinary TXM excretion has been associated with worse cardiovascular outcomes in high‐risk, aspirin‐treated non‐MPN subjects in a large phase III trial.^36^

Interestingly, the heterogeneity in platelet TXA_2_ production observed in our PV cohort was consistent with in silico simulations. The in silico modeling of patients with PV with higher TXA_2_ production required a comparable increase in the COX‐1 biosynthetic rate as in the ET condition,^9^, ^21^ whereas patients with PV with lower platelet TXA_2_ production could be modeled by the same parameters of aspirin‐treated non‐PV patients with a presumably normal bone marrow function (Figure 4).^9^ PK/PD modeling and simulation are central in informing and personalizing therapy in situations in which gaps in data are expected, such as in rare diseases,^37^, ^38^, ^39^ in case of alternative dosing regimens thus repurposing approved drugs.^37^, ^40^, ^41^ Thus, the results of the present study may help redefining the current recommendation of a b.i.d. regimen for a selected patient with PV subset (i.e., those with inadequate serum TXB_2_ suppression), rather than for the entire PV population.^42^

In the absence of PD data on the (in)adequacy of platelet inhibition by low‐dose aspirin in PV, recent therapeutic recommendations suggest a b.i.d. regimen in patients with PV with high WBC counts, history of thrombosis, and/or older age.^5^ The results of the present study suggest that the platelet count may represent an additional marker associated with incomplete platelet inhibition by low‐dose aspirin in patients with PV. Notably, in our PV cohort, WBC and platelet counts were highly correlated (Table S1). Thus, our data suggest that in patients with PV with a high hematopoietic proliferative rate, including platelet generation, a standard aspirin regimen is suboptimal despite ongoing HU and/or phlebotomy treatments.

The antiplatelet PD of aspirin may be affected by PV‐specific mechanisms. Aspirin‐inactivating esterases in plasma and erythrocytes could potentially accelerate aspirin degradation in PV.^43^ However, we could not detect any correlation between plasma esterase activity and serum TXB_2_, with age as the only independent inverse predictor of esterase activity, in agreement with previous studies in healthy subjects.^44^ Moreover, in our patients with PV, RBC counts were not associated with higher serum TXB_2_ levels.

Under physiological shear stress conditions, the vascular endothelium synthesizes PGI_2_, a vasoprotective prostanoid that downregulates platelet activation and vasoconstriction.^45^ Endothelial dysfunction in PV has been associated with erythrocytosis affecting blood viscosity and shear rate,^46^ and with endothelial expression of *JAK2V617F*.^47^ Moreover, increased inflammation^48^, ^49^ and oxidative stress^50^ have been associated with PV, and are known to contribute to platelet activation, endothelial dysfunction, and atherothrombosis in patients with cardiovascular diseases.^27^, ^51^ However, we found no correlation among markers of inflammation, oxidative stress, or PGI_2_ biosynthesis and the antiplatelet effect of low‐dose aspirin in our PV cohort.

Our study has some limitations. We did not measure the aspirin esterase activity in isolated RBCs, but given the nonsignificant association between RBC count and serum TXB_2_, a role for RBCs in degrading aspirin seems unlikely. In addition, although we tested the effect of intensifying the frequency of aspirin administration in only one patient with PV with the highest serum TXB_2_, the experimental finding of complete suppression of platelet TXA_2_ production was consistent with the in silico simulation of the clinical phenotype and prediction of its reversal to a normal pattern of platelet inhibition by a b.i.d. regimen. Some limitations apply to the in silico modeling as well. We assumed the patients with PV with serum TXB_2_less than or equal to 10 ng/ml to be close to the healthy condition and those with serum TXB_2_ greater than 10 ng/ml to be close to the ET hyper‐regenerative condition. However, we were able to perform a preliminary evaluation by using another PV dataset^23^ as the test set, and the model could predict also that previous PV dataset of patients on 40 mg q.d. aspirin (Figure 5). We have re‐calibrated the model on a dataset with serum TXB_2_ measured at a single timepoint to perform a first qualitative re‐calibration of the model to the PV condition. Importantly, the in silico predictions were consistent with the experimental data.

The results of the present study have clinical implications in demonstrating that one in three patients with PV has inadequate inhibition of platelet TXA_2_ production and TXA_2_‐dependent platelet activation, despite standard aspirin therapy. Impaired platelet COX‐1 suppression can be diagnosed with a serum TXB_2_ determination 24 h after dosing, and can be rescued by a b.i.d. dosing regimen.

## AUTHOR CONTRIBUTIONS

A.G., B.R., C.P., and E.T. designed the research and wrote the manuscript. A.G., G.P., D.H., P.R., A.D., V.C., B.P., and A.H. performed the research. G.P. and B.R. analyzed the data.

## FUNDING INFORMATION

Supported by Cancer Research UK (Catalyst Award–Aspirin for Cancer Prevention Collaboration C569/A24 to C.P. and B.R.).

## CONFLICT OF INTEREST

B.R. has received consultant and speaker fees from Bayer AG, MedScape, and Sobi. C.P. reports receiving consultant and speaker fees from Acticor Biotech, Amgen, Bayer, GlaxoSmithKline, Eli Lilly, Tremeau, and Zambon; in addition, he chairs the Scientific Advisory Board of the International Aspirin Foundation. All other authors declared no competing interests for this work.

## Supporting information


Appendix S1
