# Supplementary material for: Platelet thromboxane inhibition by low‐dose aspirin in polycythemia vera: Ex vivo and in vivo measurements and in silico simulation
Source: Clin Transl Sci. 2022 Oct 5;15(12):2958–70. doi: 10.1111/cts.13415 (PMC9747129; doi:10.1111/cts.13415)
Supplement: Supplementary file 1 — Appendix S1 [file CTS-15-2958-s001.docx]

**Petrucci, Giaretta et al.**

**Supplemental Material**

**Results**

**Patient’s characteristics**

Patients with a previous thrombosis were older than patients with no history of thrombosis (72±8 vs. 63±11 years, p=0.01), and had a significantly lower hematocrit (42.2±3.4% vs. 44.4±2.9%, respectively; p=0.02), while white blood cell (WBC), red blood cell (RBC) and hemoglobin (Hb) levels were comparable in the two sub-groups (data not shown). Patients treated with phlebotomy only had similar Hb and hematocrit levels as patients on hydroxyurea (HU), but had significantly higher leukocytes (11,890±5,179/ µL vs. 8,313±3,791/µL, respectively; p<0.001) and platelet (561,416±211,785/µL vs. 319,631±100,456/µL, respectively, p<0.001) counts, consistent with the suppressive effect of HU on multiple hematopoietic lineages.

The *JAK2-V617F* allele burden directly correlated with WBC (rho=0.51, p=0.0001) and neutrophil (rho=0.56, p=0.003) counts, as previously reported.(1) It is also known that under a healthy condition *JAK2V617F* pathway induces a complex feedback regulation from platelets to their megakaryocytes and megakaryoblasts precursors in order to maintain platelet count homeostasis.(2)

However, no significant association was observed with hematocrit, platelet count or previous thrombosis. The correlations between different hematological parameters are detailed in **Table S2**. For this reason, we didn’t insert any kind of feedback regulation in modelling the COX-1 biosynthesis rate in our model adaptation to PV condition.

**Table S1. Numerical values of major parameters in the PK/PD model.**

| Parameters | Value | Unit of measure | Original reference or data source |
| --- | --- | --- | --- |
| *Pharmacodynamics* | | | |
| *k_m_* (threshold of sigmoidal acetylation) | 17.78 | *ng^2^/ml^2^* | (3) |
| *λ* (saturation rate of the sigmoidal acetylation) | 2.4 e^-3^ (control); 14.4 (ET) | *ng/min* | (3) |
| *T_1_* (MK lifespan) | 5 (healthy); 8 (ET) | *Days* | (3) |
| *T_1_* (PV patients with serum TXB_2_ >10 ng/ml) | 8 | *Days* | (4, 5) |
| *p* (healthy subjects COX-1 biosynthesis rate) | 7.5 e^-4^ | *ng/min* | (3) |
| *f_1_* (rescaling factor of p to account for different pathological conditions) | 1 (healthy); [8-15] (ET) | [-] | (3) |
| *f_1_* (PV subjects with serum TXB_2_ <10 ng/ml) | *1.8* | *ng/min* | current work  (recalibrated from initial parameters from (3)) |
| *f_1_* (non-PV high cardiovascular subjects) | *1.5* | *ng/min* | current work  (from initial parameters in (3)) |
| *p* (PV patients with serum TXB_2_ >10 ng/ml) | *3.2* | *ng/min* | current work  (recalibrated from initial parameters in (3)) |
| *N* (platelet count factor compared to the healthy condition averaging ~ 250x10^3^/μL) | 1 (control), 6 (ET) | [-] | (3) |
| *N* (platelet count for PV patients with serum TXB_2_ <10 ng/ml) | 1.4 | [-] | current work |
| *N* (platelet count for PV patients with serum TXB_2_ >10 ng/ml) | 1.7 | [-] | current work |
| *T_3a_* | 7 (healthy); 6 (ET) | *days* | (3) |
| *Pharmacokinetics* | | | |
| k_1_ (rate from portal blood to systemic blood) | 0.943 | 1/min | (3) |
| k_2_ (rate from systemic blood to portal blood) | 0.314 | 1/min | (3) |
| k_3_ (rate from systemic blood to tissues) | 1.019 | 1/min | (3) |
| k_4_ (rate from tissues to systemic blood) | 0.398 | 1/min | (3) |
| k_he_ (hepatic extraction) | 0.943 | 1/min | (3) |
| k_d_ (Hydrolysis rate constants for tissues (k_d,T_), systemic blood (k_d,B_) and portal blood (k_d,S_) assumed to be equal, i.e., k_d,T_ = k_d,B_ = k_d,S_ = k_d_) | 0.015 | 1/min | (3) |

Parameters of COX-1 dynamics inside megakaryocytes (MKs) and platelets are intended per MK unit and platelets derived from it.

Abbreviations: ET: essential thrombocythemia; TX: thromboxane; PV: polycythemia vera.

**Table S2. Univariate correlation between clinical and haematological parameters.**

| *Parameter* | *Age* | *Sex* | *RBC* | *Htc* | *Hb* | *WBC* | *Neutrophils* | *Platelets* | *JAK burden* | *CRP* | *IL-6* |
| --- | --- | --- | --- | --- | --- | --- | --- | --- | --- | --- | --- |
| *Age* | -- | 0.07 | *-0.32* | 0.05 | 0.25 | -0.26 | -0.27 | *-0.31* | 0.05 | 0.05 | 0.21 |
| *Sex* | 0.07 | -- | 0.08 | -0.17 | 0.16 | -0.18 | *-0.31* | *0.37* | *-0.38* | 0.06 | 0.13 |
| *Erythrocytes* | *-0.32* | 0.08 | -- | *0.64* | -0.20 | *0.62* | *0.57* | 0.23 | 0.109 | -0.05 | 0.09 |
| *Hematocrit* | -0.17 | -0.18 | *0.64* | -- | *0.30* | *0.50* | *0.46* | 0.14 | 0.07 | -0.02 | 0.17 |
| *Hemoglobin* | 0.25 | 0.16 | -0.20 | *0.30* | -- | -0.21 | -0.26 | 0.05 | *-0.48* | 0.14 | 0.20 |
| *Leukocytes* | -0.26 | -0.18 | *0.62* | *0.50* | -0.21 | -- | *0.98* | *0.38* | *0.51* | -0.04 | 0.02 |
| *Neutrophils* |  |  | *0.57* |  |  | *0.96* | -- | *0.29* |  |  | 0.06 |
| *Platelets* | *-0.31* | *0.37* | 0.22 | 0.14 | 0.05 | *0.38* | *0.30* | -- | 0.05 | 0.01 | 0.01 |
| *JAK burden* | 0.05 | *-0.38* | 0.11 | 0.07 | *-0.48* | *0.45* |  | 0.05 | -- | 0.05 | 0.03 |
| *CRP* | 0.05 | 0.06 | -0.05 | -0.02 | 0.14 | -0.04 | 0.05 | 0.01 | 0.05 | -- | *0.36* |
| *IL-6* | 0.21 | 0.13 | 0.09 | 0.17 | 0.2 | 0.02 | 0.06 | 0.01 | 0.03 | *0.36* | -- |

Correlation analysis according to Spearman. Significant correlations are indicated in italics.

Abbreviations: CRP: C reactive protein; IL: interleukin.

**Table S3. Characteristics of PV patients with sTXB_2_ values below and ≥ 10 ng/ml.**

| *Variable* | *<10 ng/ml* | *≥10 ng/ml* | *P value* |
| --- | --- | --- | --- |
| *Erythrocytes, x10^6^/uL* | 4.74 [3.298.-8.078] | 6.15 [3.160-7.780] | 0.05 |
| *Hemoglobin, g/dL* | 14 [12.6-15.6] | 13.4 [11.5-17.3] | 0.57 |
| *Hematocrit, %* | 44.2 [37-48.4] | 44 [36-50] | 0.52 |
| *Leukocytes, /uL* | 7,340 [3542-19568] | 8,755 [5330-21500] | 0.08 |
| *Neutrophils, /uL* | 4,725 [2373-16081] | 6,326 [3528-19135] | 0.07 |
| *JAK2V617F burden* | 55 [5.7-92] | 58.9 [5.8-97.3] | 0.44 |
| *Disease duration, years* | 3 [1-9] | 5 [1.75-9.7] | 0.34 |
| *Plasma esterase, uM/L/min* | 53 [35-147] | 50 [30-183] | 0.49 |
| *hs-CRP* | 1.23 [0.8-2.5] | 0.9 [0.6-3.1] | 0.73 |
| *Interleukin-6* | 4.6 [2.5-11.3] | 3.7 [0.001-8.6] | 0.28 |
| *urinary PGIM, pg/mg creatinine* | 207 [56-631] | 159 [50-485] | 0.40 |
| *8-iso PGF_2a_, pg/mg creatinine* | 719 [113-1557] | 805 [369-2456] | 0.19 |

Abbreviations: CRP: C reactive protein; PGIM: prostacyclin metabolite; PG: prostaglandin.

**Table S4. Haematological values and their correlations of 8 PV patients who were studied in two repeated occasions.**

| *Variable* | *Visit 1* | *Visit 2* | *rho* |
| --- | --- | --- | --- |
| *Erythrocytes x10^6^/uL* | 5.23±0.85 | 5.16±1.24 | 0.52 |
| *Hemoglobin, g/dL* | 14.1±0.9 | 14.2±1.2 | 0.39 |
| *Hematocrit, %* | 44.6±1.7 | 44.1±3.3 | 0.18 |
| *Leukocytes /uL* | 7,750±1,382 | 7,317±1,158 | *0.75** |
| *Platelets /uL* | 370,500±170,937 | 360,250±174,804 | *0.93** |

*p<0.01

**Table S5. Comparison between data and model predictions.**

| *Condition*  *(corresponding Figure)* | *Measured TXB_2_ %*  *Mean ± standard deviation* | *Predicted TXB_2_ % for the average patient* | *25^th^-75^th^ percentile interval of measured TXB_2_*  *% of baseline* |
| --- | --- | --- | --- |
| Non-PV high cardiovascular risk subjects (Fig.4A) | 0.93 % ± 0.54 %  (Fig.4A average value in the green bar) | 1.40 %  (Fig.4A purple line) | 0.59-1.42 |
| PV subjects  TXB_2_ <10 ng/ml cutoff  (current set) (Fig.4A) | 0.88 % ± 0.45 %  (Fig.4Aaverage value in the red bar) | 1.07 %  (Fig.4A blue line) | 0.59-1.32 |
| PV subjects  TXB_2_>10 ng/ml cutoff  (current set) (Fig.4B) | 4.45 % ± 3.28 %  (Fig.4B average value in red bar) | 5.24 %  (Fig.4B blue line) | 2.26-5.39 |
| PV subjects  TXB_2_ >10 ng/ml cutoff  (old set) (Fig.5 orange line) | 13.04 % ± 20.21 %  (Fig.5 average value in green bar) | 9.61 %  (Fig.5 orange line) | 4.30-18.65 |

Abbreviations: PV: polycythemia vera; TX: thromboxane.

**References**

(1) Larsen, T.S., Pallisgaard, N., Moller, M.B. & Hasselbalch, H.C. The JAK2 V617F allele burden in essential thrombocythemia, polycythemia vera and primary myelofibrosis--impact on disease phenotype. *Eur J Haematol* **79**, 508-15 (2007).

(2) Koride, S., Nayak, S., Banfield, C. & Peterson, M.C. Evaluating the Role of Janus Kinase Pathways in Platelet Homeostasis Using a Systems Modeling Approach. *CPT Pharmacometrics Syst Pharmacol* **8**, 478-88 (2019).

(3) Giaretta, A., Rocca, B., Di Camillo, B., Toffolo, G.M. & Patrono, C. In Silico Modeling of the Antiplatelet Pharmacodynamics of Low-dose Aspirin in Health and Disease. *Clin Pharmacol Ther* **102**, 823-31 (2017).

(4) Malherbe, J.A. *et al.* Dysregulation of the intrinsic apoptotic pathway mediates megakaryocytic hyperplasia in myeloproliferative neoplasms. *J Clin Pathol* **69**, 1017-24 (2016).

(5) Prins, D. *et al.* The stem/progenitor landscape is reshaped in a mouse model of essential thrombocythemia and causes excess megakaryocyte production. *Sci Adv* **6**, eabd3139 (2020).
